# A multi-camera and multimodal dataset for posture and gait analysis

**DOI:** 10.1038/s41597-022-01722-7

**Published:** 2022-10-06

**Authors:** Manuel Palermo, João M. Lopes, João André, Ana C. Matias, João Cerqueira, Cristina P. Santos

**Affiliations:** 1 CMEMS–UMinho, University of Minho, Guimarães, Portugal; 2 LABBELS–Associate Laboratory, Braga/Guimarães, Portugal; 3 Clinical Academic Center (2CA-Braga), Hospital of Braga, Braga, Portugal; 4 Life and Health Sciences Research Institute (ICVS), University of Minho, Braga, Portugal

**Keywords:** Biomedical engineering, Rehabilitation

## Abstract

Monitoring gait and posture while using assisting robotic devices is relevant to attain effective assistance and assess the user’s progression throughout time. This work presents a multi-camera, multimodal, and detailed dataset involving 14 healthy participants walking with a wheeled robotic walker equipped with a pair of affordable cameras. Depth data were acquired at 30 *fps* and synchronized with inertial data from Xsens MTw Awinda sensors and kinematic data from the segments of the Xsens biomechanical model, acquired at 60 Hz. Participants walked with the robotic walker at 3 different gait speeds, across 3 different walking scenarios/paths at 3 different locations. In total, this dataset provides approximately 92 minutes of total recording time, which corresponds to nearly 166.000 samples of synchronized data. This dataset may contribute to the scientific research by allowing the development and evaluation of: (i) vision-based pose estimation algorithms, exploring classic or deep learning approaches; (ii) human detection and tracking algorithms; (iii) movement forecasting; and (iv) biomechanical analysis of gait/posture when using a rehabilitation device.

## Abstract (summary)

**Table:** 

Measurement(s)	depth images • kinematic data
Technology Type(s)	RGB-D camera • Inertial Motion Capture System
Sample Characteristic - Organism	Homo Sapiens

## Background & Summary

According to the World Health Organization, nearly 15% of the World’s population suffers from some form of disability, arising to 1 billion^1^, being dysfunctional gait a common disability, especially in Europe, where was estimated that 5 million persons depend on a wheelchair^2^. This results from an aging population, but also due to the global incidence of cardiovascular and/or neurological disorders, such as cerebellar ataxia following a stroke, cerebral palsy, or, among others, Parkinson’s disease^3–5^. These disorders may result in cognitive impairments, as well as lack of stability, affected motor coordination, and muscle weakness, leading to an increased risk of falls and fall-related injuries^2^. Consequently, quality of life is highly jeopardized, causing social-economic consequences due to the increased institutionalization and dependence on others^6,7^.

Robotics-based rehabilitation is an evolving area that aims to improve the quality of life of motor-impaired persons by providing residual motor skills recovery based on repetitive and intensity-adapted training along with assistive devices^2^. In rehabilitation, human motion analysis (namely gait and posture) is relevant as it allows the assessment of joint kinematics, enabling the evaluation of spatial and temporal parameters^8^, that may enable the design of more user-centred approaches considering the person’s disability level and enables to assess the patient’s evolution throughout therapy^9,10^. Furthermore, human motion analysis, and particularly gait analysis, can also be an important tool in surgery since it allows to choose the most judicious surgical treatment to apply according to the gait pattern^8^.

Current solutions for human motion analysis are normally based on optical motion capture (MoCap) systems with retro-reflective markers, such as Vicon (Vicon Motion Systems, UK) or Qualisys (Qualisys AB, Göteborg, Sweden). Although accurate and considered a gold standard, these systems require complex setups along with specific environments and workspaces^11^. Other optical solution, less expensive and without the need of markers, *e.g*. Kinect (Microsoft Corporation, USA), has been presented in literature^12,13^. However, this solution is susceptible to errors when compared to marker-based optical MoCap systems, presenting poor validity regarding gait kinematic variables^13^, especially on the feet and ankle joints^14^. Still, it was considered valid for some spatiotemporal parameters of gait^13^. Inertial-based MoCap systems were also presented in literature to measure joint kinematics, being also a less expensive solution than marker-based optical MoCap systems and, considering that are based on Inertial Measurement Units (IMU), these systems are wearable and can be used outside laboratory contexts, more specifically in clinical ambulatory settings^11^. These were found to be suitable for human motion analysis, although presenting challenges inherent to drift associated to numerical integration of angular rate measurements and ferromagnetic disturbances when using magnetometers^11,15^.

Recent studies involving vision-based machine learning techniques are showing great potential for human motion analysis, aiming accurate 3D pose estimation. Besides being a less expensive solution, evidence shows reasonable precision on estimating the person’s pose without the need of wearable markers/sensors nor complex setups^16^. Nevertheless, this approach requires a considerable amount of quality data to train the models and achieve the precision and accuracy required to be an effective human motion analysis tool. Furthermore, these algorithms need to be validated in real world scenarios considering the final application.

Available datasets in literature present footage of general activities, including daily life activities, sports movements, and general locomotion. Examples include Human3.6 M^17^, TotalCapture^18^, MoVI^19^, MPI-INF-3DHP^20^, and Panoptic^21^. These datasets present 3D kinematics, obtained with retro-reflective markers^17–19^, IMU^18,19^, and markerless MoCap systems^20,21^. However, none of these dataset present camera-related data and 3D kinematics along with robotic assistive devices, namely robotic walkers, which are relevant to assess the biomechanics of gait and posture when using such devices, and to correlate data with that acquired with the device to develop pose estimation algorithms, for instance. Additionally, data are normally acquired within dedicated workspaces^17–19,21^, within controlled conditions, and with non-moving cameras, which do not capture real-world scenarios.

To attain these challenges, we present in this study a multi-camera vision dataset involving 14 healthy participants walking with WALKit Smart Walker, a personalized and user-oriented robotic walker for ataxic gait and posture rehabilitation^7^. Vision data were acquired with the smart walker embedded cameras together with inertial-based data acquired with the commercially available Xsens MTw Awinda MoCap system^22^. This system was used as the ground truth of kinematic data and to bring data collection closer to a clinical setting, allowing data to be acquired outside a laboratory environment. The dataset includes inertial data from MTw sensors, kinematic data of the segments, and depth frames of both upper and lower body, captured with the smart walker moving cameras. Data were collected considering different environment-contexts and slow gait speeds (0.3, 0.5, and 0.7 m/s), typical of persons with motor disabilities^23^. This dataset distinguishes itself from others by providing multimodal data, with motion capture pose information, on dynamic environments with people walking by, approaching the real environment of clinical facilities, and with a robotic walker that integrates non-overlapping cameras in movement. To the best knowledge of the authors, this is the first vision-based dataset involving the capture of upper and lower body depth frames for pose estimation with a robotic smart walker.

The proposed dataset may contribute to further assessment, monitoring, and rehabilitation of persons with motor disabilities, allowing the development and evaluation of (**i**) classic and deep learning vision-based pose estimation algorithms; (**ii**) applications in human detection and joint tracking, (**iii**) applications in movement forecasting, and (**iv**) methods for the biomechanical analysis of gait/posture when using a rehabilitation device.

## Methods

### Participants

Healthy participants from the academic community of the University of Minho were contacted to participate in the study. They were provided with the study details, namely the inclusion criteria, protocol, and duration. The participants were recruited and selected based on a set of inclusion criteria, as follows: (**i**) present healthy locomotion without any clinical history of abnormalities; (**ii**) present total postural control; (**iii**) present body height between 150 and 190 cm, and (**iv**) have 18 or more years old.

Considering these statements, 14 healthy participants (10 males and 4 females; body mass: 69.7 ± 11.4 kg; body height: 172 ± 10.2 cm; age: 25.4 ± 2.31 years old) were recruited and accepted to participate, voluntarily, in the data collection (Table 1). All participants provided their written and informed consent to participate in the study, according to the ethical conduct defined by the University of Minho Ethics Committee (CEICVS 147/2021) that follows the standard set by the declaration of Helsinki and the Oviedo Convention. Participants’ rights were preserved and, as such, personal information that could identify them remained confidential and it is not provided in this dataset.

**Table 1:** Participants’ main anthropometric data.

Participant_ID	Gender (M/F)	Age (years)	Body mass (kg)	Body height (cm)
00	M	23	63	180
01	F	24	51	151
02	M	22	89	185
03	F	28	70	159
04	F	27	53	157
05	M	30	68	172
06	M	28	75	185
07	M	24	86	170
08	M	24	73	170
09	M	26	84	175
10	M	23	64	172
11	M	26	64	175
12	M	26	74	182
13	F	24	63	171
Average (±std)	4 females, 10 males	25.4 (±2.31)	69.7 (±11.4)	172 (±10.2)

More anthropometric information can be found on the dataset’s metadata file.

### Participants instrumentation

**Fig. 1:**
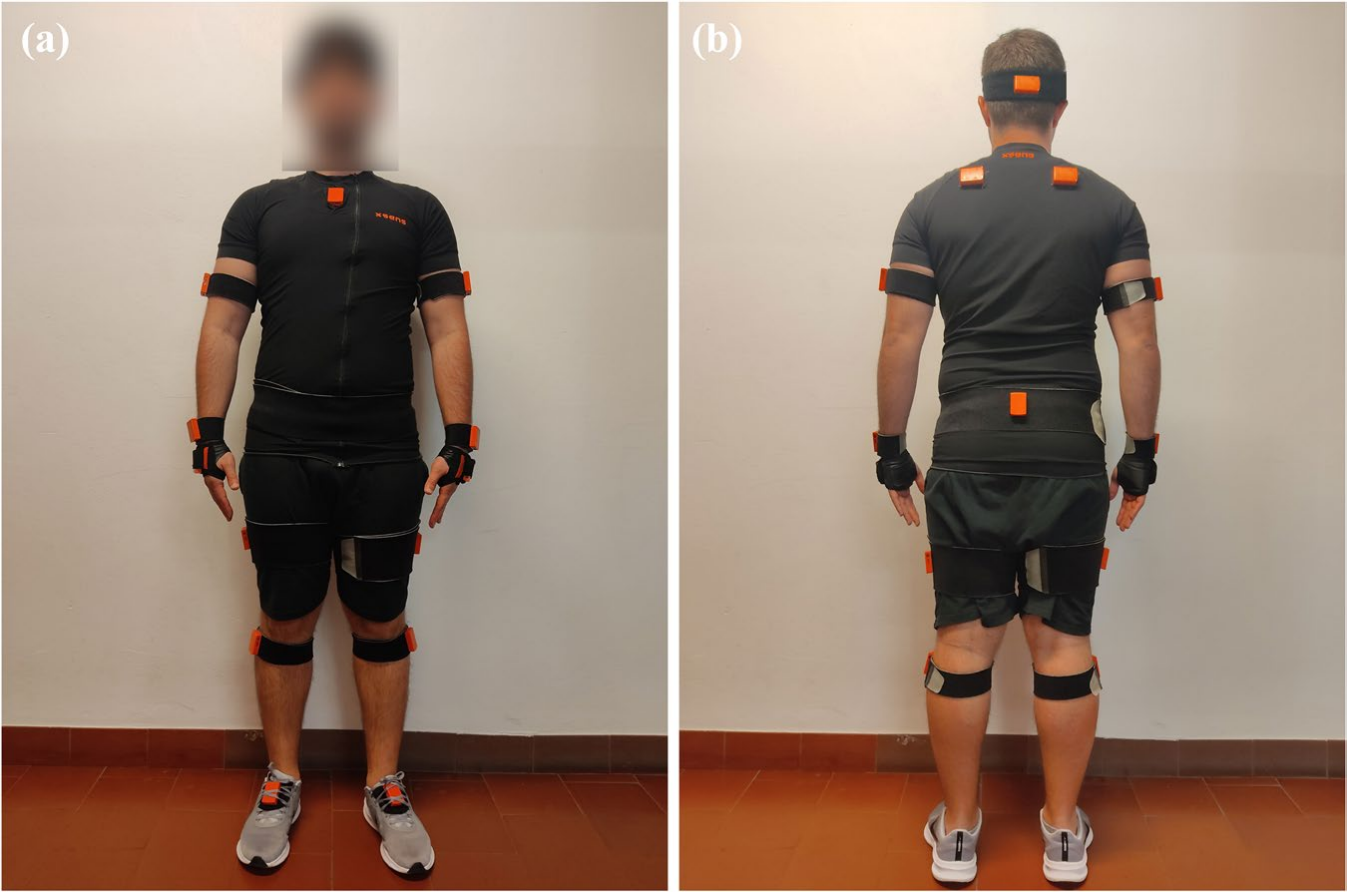
Inertial sensors placement: (**a**) participant’s anterior view, and (**b**) participant’s posterior view. Note that each sensor was placed in the inner of the strap to better secure the sensors. Additionally, the *sternum*, head, and foot sensors were placed inside the frontal pocket of the Xsens suit, on the pocket of the headband and inside each shoe, respectively.

Each participant wore the full-body inertial motion tracking system MTw Awinda (Xsens Technologies, B.V., The Netherlands, validated in^15^), as illustrated on Fig. 1, placing seventeen IMUs on head, both shoulder, *sternum*, upper arms, forearms, wrist, pelvis, upper leg, lower leg, and feet. Since this device measures orientation, and not position, the precision of the sensors’ position is not very relevant, although these were placed as much as possible as the recommendation^24^. Moreover, when performing the calibration, the orientation of each sensor will align with the orientation of each segment of the Xsens biomechanical model^24^, removing the operator bias. Nevertheless, the sensors’ placement was performed by the same researchers, ensuring repeatability in the instrumentation procedure.

### WALKit smart walker

**Fig. 2:**
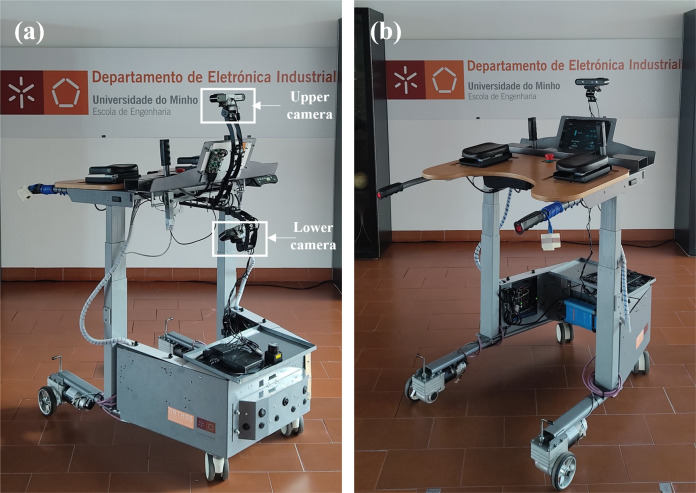
Robotic walker used to collect this dataset, considering **(a)** the frontal view and **(b)** the rear view. Both upper and lower cameras are highlighted in (**a**) with a white box.

Each participant used WALKit Smart Walker, as illustrated in Fig. 2. This robotic device is a four wheeled walker with two motors on the rear wheels and two caster-wheels at the front^25^. It integrates multiple sensors, namely two Orbbec Astra RGB-D cameras (Orbbec 3D Technology International Inc., USA), a laser range finder sensor (URG-04LX, Hokuyo Automatic Co., Ltd, Japan), 9 ultrasonic sensors (LV-MaxSonar-EZ, MaxBotix Inc., USA), an infrared sensor (GP2Y0A21YK0F, Sharp Corporation, Japan), two load cells (CZL635, Phidgets Inc., Canada), and an external IMU (MPU-6050, InvenSense, USA) to be used by the user. The cameras present complementary fields of view: the upper camera records the user’s trunk, and the lower camera records both legs and feet. All data provided by these sensors, as well as the functionalities related to them, can be accessed by both patient and clinician, using a dedicated LCD touch screen. This device presents a hierarchical control divided into low- and high-level. The low-level runs a real-time operating system (RTOS) on an STM32F4 Discovery and it is responsible to acquire data from all sensors, with exception of both cameras and the laser range finder, and send this information to the high-level. Additionally, this control level is responsible to read the user’s motion commands, expressed with an intuitive handlebar that moves to the front or sides, and convert this into reference velocity commands of a Proportional-Integral-Derivative (PID) controller. By the other side, the high-level control runs a Robot Operating System (ROS) on a minicomputer. This level is responsible to process all the sensors’ information sent by the low-level and to implement different functionalities with this. Additionally, the high-level control is also responsible to interpret motion commands sent by an external person while using a remote controller, which may be relevant in early stages of therapy in which patients do not have sufficient coordination to control the device.

### Data collection

Data collection was performed in the School of Engineering of University of Minho. Data collection included: (**i**) inertial data from the MTw sensors, namely 3D free acceleration (*i.e.*, without the gravitational component), orientation and magnetic field, which were measured at 60 Hz; (**ii**) kinematic data of the segments, namely 3D segment acceleration, angular velocity, angular acceleration, global position, and velocity, which were calculated by the MVN Analyze; **iv**) kinematic data of the body’s joints, namely the joint’s angles, also calculated by the MVN Analyze; and **v**) depth images from the walker’s embedded cameras captured at 30 frames-per-second (*fps*) with a resolution of 640 × 480 pixels. Note that, although the cameras allow the collection of RGB-D data, this dataset only provides depth images, following considerations related to patients’ privacy. All data were timely synchronized using a software trigger. More details regarding data synchronization can be found in the Technical Validation Section.

### Experimental protocol

After the placement of the MTw Awinda sensors, the required participants’ anthropometric data were measured according to the Xsens guidelines^26^. These dimensions were introduced on the MVN Analyze software to adjust the biomechanical model to the participant. Segment calibration followed the manufacturer’s guidelines, which is a required step to align the motion trackers with the participants’ segments. Each participant assumed the *N-pose*, which refers to a neutral position of segments as illustrated in Fig. 1a,b. The participants held this position for four seconds, and then walked forward, turned, and walked backwards in a normal fashion. Once the participants reached the initial position, they assumed the *N-pose* position again. During this step, each participant held a stick with an additional IMU (PROP sensor) to set up this sensor, as indicated by Xsens. After the calibration, this additional IMU was placed on the walker’s upper camera in order to provide its orientation regarding the MVN global axis.

Subsequently, each participant experienced a one-day protocol in which they performed 3 trials, one per each slow gait speed (0.3, 0.5, and 0.7 m/s), which were considered since these are often observed in persons with motor disabilities^23^, and considering 3 different sequences: (**i**) walking forward in a corridor for about 10 meters, (**ii**) turning right in a corner, and (**iii**) turning left in a corner. Each trial was repeated 3 times for better statistical significance during movement evaluation, but in different locations, to accommodate different scenarios and environment conditions, approaching the real environment of clinical facilities. Note that each participant performed the same three conditions.

Each trial was segmented into three steps, as follows: step 1 - the walker was placed on the starting line of each location (these were measured and drawn on the floor prior to data collection); step 2 - the participants were placed in front of the walker, and were asked to assume the *N-Pose* to reset the IMUs internal referential; and step 3 - the participants were asked to grab the two handles of the walker. After these first three steps, data collection started using a remote controller, which was used by the researcher to guide the walker and to send a digital pulse to start recording synchronously both cameras and the MVN software. The participants walked normally until they reached the end line of the trial. Finally, the recording was stopped using the remote controller and the walker was moved to the next trial’s starting location, repeating the process. Prior to data collection, the participants performed a familiarization trial with the robotic walker and the selected gait speeds.

### Dataset elaboration

#### Raw data

For each individual trial, data obtained from the MTw Awinda was reprocessed by the MVN Analyze software, and then exported to *“.csv”* and *“.c3d”* formats. These were selected since: (**i**) the *“.csv”* contains a complete set of information, from raw IMU sensor data during acquisition to segment positions in 3D space and joints’ angles; and (**ii**) the *“.c3d”* files contain a more complete point set in 3D space and is a common standard in biomechanics and gait analysis. The joints/points contained on the files exported from the MVN Analyze can be found on the MVN user manual^27^.

The depth frames were saved as individual *“.png”*, with pixel values corresponding to the distance regarding the camera’s sensor, in millimeters. This file was saved with 16-bit precision, to avoid loss in the depth information, which also prevented encoding the frames to video format.

The above mentioned data is referred to as “raw data”, as it only received the processing necessary to actually be used outside the respective acquisition software/hardware.

#### Calibration data

A set of data was required to obtain the relationship between the cameras’ position and orientation (*i.e*., the transformation matrix). These data is referred to as “calibration data”.

A referential transformation between the walker’s cameras was obtained by using a checkerboard visible from both cameras and performing stereo calibration, which allows to determine the relative geometry between cameras, namely rotation and translation^28^. Since both cameras only overlap about 3 meters away from the walker and on a narrow strip of the image, available stereo calibration methods, presented in OpenCV, performed poorly. For this reason, an alternative method was used. Firstly, the 2D coordinates of the checkerboard corners were detected in the camera’s RGB frames by using the OpenCV library^29^. Secondly, these points were projected to 3D coordinates considering the depth information. Lastly, the affine transformation between the 3D coordinates with the lowest re-projection error was found, using the RANSAC algorithm^29,30^.

A translation between the upper camera and the tip of each handle of the walker was also found to later relate the skeleton 3D coordinates with the cameras’ information. This was obtained by using a stick with an ArUco marker (which is a fiducial marker^31^ that can be used as a point of reference in an image), whose tip was placed on the desired handle position, rotated over multiple frames, creating a virtual sphere. The tip position relative to the upper camera was found by solving a system of equations for the center of the created sphere.

#### Processed data

Data from the inertial motion tracking and the walker’s depth images are synchronized temporally using timestamps which were recorded during acquisition with the walker’s embedded software. The corresponding temporal indexes for each data modality were saved in a *“.csv”* file which can be used to easily select data when needed, while also keeping all raw samples obtained.

The 3D joint data obtained from the MVN Analyze uses the global axis referential where the MVN character moves around as the person moves and rotates with the walker. As an optional processed data, the skeleton position was normalized to the origin of the global axis, considering the center-of-mass position, and the heading was removed. In this way, the biomechanical model is always facing forward, which may be relevant for applications in which the user’s orientation regarding the global axis is not relevant. This processed data is referred to as *“normalized_skeleton_3D”*.

**Fig. 3:**
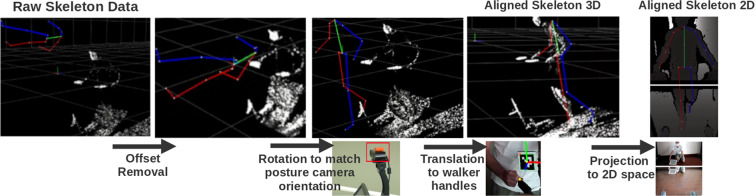
Transformations to spatially align the Xsens skeleton data from a world referential (left) to the walker’s posture camera referential (right). Transformations are shown in 3D space along with the 3D point cloud for reference. Once the skeleton is aligned in 3D space, it is possible to project it to the cameras’ referential.

A more complex method to relate the joints’ positions with the walker’s cameras was also performed. It is summarized in Fig. 3. This method transforms the 3D data of the biomechanical data from the MVN global axis to the cameras’ referential. First, the skeleton root joint was centered in the referential origin. Then, a rotation was applied that transforms the referential of the skeleton to the referential of the upper camera. This rotation is obtained from the additional MTw Awinda PROP sensor placed over the upper camera during data acquisition, as previously stated. Lastly, a translation was applied to place the skeleton’s wrists in the same position as the corresponding walker’s handles. This offset was obtained in the extrinsic calibration step, as explained in the “Calibration data” subsection and whose validity is detailed in the “Technical Validation” section. This method is valid as long as the participant is always grabbing the walker’s handles, which was ensured during acquisition.

This transformation could be computed for only one of the hands, as both should give the same result. However, the average value of both transformations was used to reduce symmetrical errors coming from the Xsens calibration procedure. This processing steps allowed having labeled 3D joints which are spatially related to the information obtained from the cameras’ data. These data is referred to as *“aligned_skeleton_3D”*.

Once the skeleton 3D coordinates were relative to the referential of the upper camera, it was possible to project the joint positions to 2D space, using the camera intrinsic parameters, which were used to label the joints in the depth frames. This projection was direct in the frames of the upper camera, but for lower camera it was first necessary to apply an extrinsic transformation which converted the points from the upper camera to the lower. These data is referred to as *“aligned_skeleton_2D”*.

Since the dataset contains the depth information, it is straightforward to obtain a fused colored point cloud with data from both cameras. This involves projecting the depth frames to 3D space using the camera intrinsic parameters for each of the cameras, then applying a referential transformation to transform the gait data point cloud into the upper camera referential. These data are not being saved as part of the *“processed_data”* since it occupies a significant amount of space and can be obtained later if needed through the scripts that accompany this database.

Additionally, the feet joints from the skeleton contained in the *“Segment Position.csv”* file (“foot”, “toe”) were, in all methods, replaced with the ones from the *“.c3d”* file (“heel”, “toe”). This moves the foot keypoints from the ankle to the heel, which is more relevant for the analysis of gait metrics^7^.

## Data Records

**Fig. 4:**
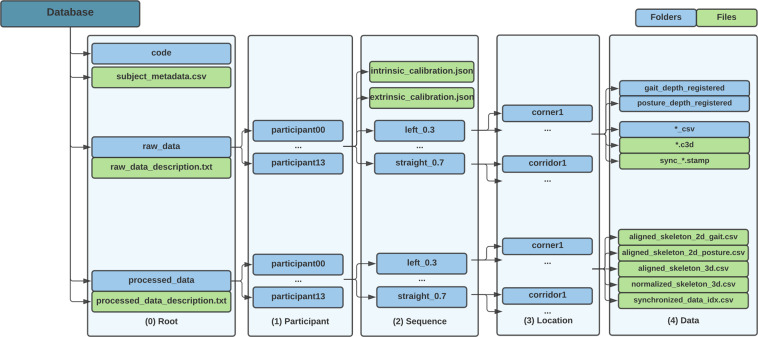
Hierarchical folder structure of the database.

All data files are available online on a *PhysioNet* database^32^. This dataset is structured hierarchically, providing an intuitive and easy way to select the data. It is organized in 5 levels, as illustrated in Fig. 4, as follows: (**i**) level 0: **Root**, includes participant’s metadata, general dataset information, raw data folders, and processed data folders; (**ii**) level 1: **Participant**, includes a folder for each of the fourteen participants of this data collection; (**iii**) level 2: **Sequence**, contains a folder for each performed sequence (walking straight or turning and its speed), along with both intrinsic and extrinsic calibration files; (**iv**) level 3: **Location**, includes a folder with the repetition’s location ID (corner1/2/3 and corridor1/2/3); and (**v**) level 4: **Data**, presents the data files for each of the aforementioned modalities.

### Raw data

Raw data are provided not only to replicate results, but also to allow users to parse it in alternative ways, enabling further extraction of relevant information. These data are organized hierarchically inside the *“raw_data”* folder (see Fig. 4, level 0), following the previously detailed structure. Raw data includes: (**i**) calibration data, with both intrinsic and extrinsic files; (**ii**) the skeleton joint data obtained with the MVN Analyze software; (**iii**) the cameras’ depth frame data; and (**iv**) a synchronization file (*“.stamp”*). The synchronization file corresponds to the instant the trigger signal was sent to the MTw Awinda base station to start recording. This was necessary since the walker’s high-level software, which is based on ROS, caused some delay (~0.65 sec) when acquiring the depth frames. Nevertheless, this delay is not considered relevant since the user was instructed to start the protocol after both devices are recording, and this synchronization file allowed the data to be aligned offline.

#### Calibration data

Inside each of the participants’ directories (Fig. 4, level 2), two calibration files are presented: one for the cameras’ intrinsic parameters, and another for the extrinsic referential transformations that allows both stereo calibration between the two cameras and the positioning of the biomechanical model regarding the walker’s upper camera. These files were respectively named *“intrinsic_calibration.json”* and *“extrinsic_calibration.json”*.

#### Skeleton joint data

Two groups of files obtained from the MVN software are presented in level 4 for each individual trial. These include: (**i**) exported *“.csv”* files from the MVN Analyse software, and (**ii**) exported *“.c3d”* files also processed by the MVN software, containing a more complete set of body keypoints extrapolated from the biomechanical model. This format is also commonly used in biomechanical analysis^33^.

Regarding the *“.csv”* files, these include 16 different files, containing: (**i**) a full set of inertial data for the sensors in x-, y-, and z-axis, including free acceleration (*“Sensor Free Acceleration”*, expressed in [*m/s*^2^], without the gravitational component), orientation both in Euler and quaternion (*“Sensor Orientation - Euler”*, expressed in [*deg*], and *“Sensor Orientation - Quat”*, respectively), and magnetic field (*“Sensor Magnetic Field”*, in [*a.u*.]); (**ii**) the segments’ kinematic data in in x-, y-, and z-axis expressed in the global frame, including acceleration (*“Segment Acceleration”*, expressed in [*m/s*^2^]), angular velocity (*“Segment Angular Velocity”*, expressed in [*rad/s*]), angular acceleration (*“Segment Angular Acceleration”*, expressed in [*rad/s*^2^]), orientation - euler/quaternion (*“Segment Orientation - Euler”* and *“Segment Orientation - Quat”*, respectively), position (*“Segment Position”*, expressed in [*m*]), and velocity (*“Segment Velocity”*, expressed in [*m/s*]); (**iii**) the joint angles considering sequence *XZY* and *ZXY* (*“Joint Angles XZY”* and *“Joint Angles ZXY”*, respectively) expressed in [*deg*]; (**iv**) the center-of-mass position (*“Center of Mass”*), expressed in [*m*]; and (**v**) the ergonomic joint angles, which are a list of specific joints used in ergonomic analysis, considering sequence *XZY* and *ZXY* (*“Ergonomic Joint Angles XZY”* and *“Ergonomic Joint Angles ZXY”*, respectively) expressed in [*deg*]. For each file, each joint/segment has its own column, with the samples listed in the rows. More details regarding the anatomical model can be found in the Xsens MVN manual^27^.

#### Cameras’ frame data

Depth frames from each of the cameras were saved into the respective folders (*“gait_depth_registered”* and *“posture_depth_registered”*). These data can not be converted into video format, as no *codec* that correctly supports the 16-bit precision was found. A timestamp was also saved for each of the depth frames, and was written in the name of each file.

### Processed data

All the processed data is stored inside the *“processed_data”* folder (see Fig. 4, level 0) and follows the same hierarchical structure as the *“raw_data”* folder. The files for each trial are organized in level 4. These data allows reading and using the dataset more easily and with minimal dependencies from the previous pre-processing steps. It is composed of 5 files saved in *“.csv”* format. Four of them contain the joint data obtained through the methods described on the “Methods - Process data” Section, and were saved on the corresponding folders, namely: the normalized joint data in 3D space (*“norm_skeleton_3d.csv”*, expressed in [*m*]), the aligned joint data in 3D space (*“aligned_skeleton_3d.csv”*, expressed in [*m*]), and the aligned 2D joint data for the lower (*“aligned_skeleton_2d_gait.csv”*) and upper (*“aligned_skeleton_2d_posture.csv”*) cameras, expressed in pixels. The first column of each file corresponds to the number of samples, and the following correspond to a joint, namely pelvis, 5*th* lumbar spine (L5), 3*rd* lumbar spine (L3), 8*th* and 12*th* thoracic spine (T8 and T12, respectively), neck, head, right/left shoulders, right/left upper arms, right/left forearms, right/left hands, right/left upper leg, right/left lower leg, right/left foot, and right/left toe. Each line corresponds to a sample. It should be noted that in the case of the 2D data, some of the points are projected outside the image frame as they are not seen by the camera sensor, however their position in the 2D camera plane is still valid.

An additional file was added (*“synchronized_data_idx.csv”*), containing indexes of corresponding data samples for each modality, in order to synchronize the processed data samples with the video and depth files which are stored raw, as obtained from the walker. In this file, the first column corresponds to the number of temporally aligned samples, and the following correspond to the aligned frame of both upper (*“depth_posture_idx”*) and lower (*“depth_gait_idx”*) cameras, and the corresponding Xsens sample (*“xsens_idx”*).

### Metadata

Metadata were collected from all participants. These include (**i**) age, (**ii**) gender, (**iii**) body mass, (**iv**) body height, and (**v**) body dimensions, namely: hip height, shoe length, shoulder height, shoulder width, elbow span, wrist span, arm span, hip width, knee height, and ankle height. This information is stored on *“subjects_metadata.csv”* file which was placed on the root folder location (Fig. 4, level 0). Additionally, information regarding the organization and data contained in the *“raw_data”* and *“processed_data”* folders is also presented in two data description files (*“raw_data_description.txt”* and *“processed_data_description.txt”*, Fig. 4, level 0).

### Data limitations

During the dataset organization, we observed some data irregularities that should be considered when using this dataset. A few trials were discarded due to sensor displacement during a sequence or file corruption on some of the modalities when processing. These trials amount to 15 of the initial 378 (≈4%) and are enumerated on Table 2.

**Table 2:** List of discarded trials.

Trial	Observation
participant05_left_0.3_corner1	Loose Xsens sensor
participant05_right_0.5_corner2	Incorrect Xsens data
participant05_straight_0.5_corridor1	Incorrect c3d data
participant05_straight_0.5_corridor2	Incorrect c3d data
participant05_straight_0.5_corridor3	Incorrect c3d data
participant05_straight_0.7_corridor1	Incorrect c3d data
participant05_straight_0.7_corridor2	Incorrect c3d data
participant05_straight_0.7_corridor3	Incorrect c3d data
participant08_left_0.3_corner3	Loose Xsens sensor
participant08_left_0.5_corner3	Loose Xsens sensor
participant08_straight_0.3_corridor1	Invalid depth data
participant09_right_0.3_corner3	Loose Xsens sensor
participant09_straight_0.3_corridor3	Loose Xsens sensor
participant09_straight_0.5_corridor3	Loose Xsens sensor
participant09_straight_0.7_corridor3	Loose Xsens sensor

Each trial was initiated with the walker stopped, and thus will contain a variable initial number of frames which do not correspond to normal walking dynamics, usually in the first second of each trial. Additionally, in some trials, the depth data from the walkers’ cameras was partially affected from infrared exposure from sunlight. In both cases, these data were considered representative of real environment variability, found in real sessions.

Additionally, we identified some limitations of the proposed dataset. It contains limited variability in terms of walking patterns, since we focused on level-ground walking with healthy participants. Therefore, no data containing abnormal walking patterns are presented in this dataset. Moreover, the MVN joint locations are highly dependent on the biomechanical model and body dimensions taken from the participant, and may present some deviations regarding the physical position on the human body. This was mostly visible on the hip joints.

Another limitation concerns to the *aligned_skeleton* data. Although providing reasonable estimates of the human joint locations, these data are affected by compounding transformation errors which add to the MVN Awinda intrinsic error. This might produce lower quality alignments between the visual data and the joint data. This was minimized as much as possible in the protocol. Nevertheless, if camera-relative positional data is not necessary, then the normalized skeleton may be used, as it is not affected by these errors.

## Technical Validation

### Data acquisition

In order to ensure the quality of the data produced, the participants were asked to follow the established protocol, while being supervised and guided by the main researcher. Before starting the trial acquisition, the Xsens MTw Awinda MoCap system was calibrated following the MVN Analyze instructions. This was performed for each participant. Then a real-time visualisation of the MVN character was performed to check if it reacted according to the participant’s movement, which was confirmed. During the trials, the participants were asked to interact with the device, while maintaining their normal gait pattern for the gait speed imposed by the device, to reduce bias in the movements produced.

### Data synchronization

For data synchronization, we used the Xsens MTw Awinda base station in configuration to receive a trigger to start recording. This trigger was sent by the robotic walker, using the low-level control. Due to ROS latency, the timestamp in which the start trigger was sent was recorded, which is provided in the *“.stamp”* file of raw data level, and each timestamp of each depth frame acquired with the two Orbbec Astra cameras were also recorded. The temporal synchronization was ensured by matching offline each timestamp of the depth frame, acquired at 30 *fps*, with the corresponding sample of the Xsens MTw Awinda through the function “align_data_by_timestamp” from *utils.py*, which is provided in this dataset. Although we have followed this protocol to ensure data synchronization, this is not error-free, thus we suggest users to check it before using data.

### Data projection to 2D and 3D space

The projection error was verified considering the transformation error between both cameras and the translation from the walker handles to the upper camera. These were validated using the OptiTrack V120:Trio (NaturalPoint, Inc., Oregon, USA). For the first case, a translation error of 2.53 cm was obtained, whereas an error of 1.99 cm was verified for the second case.

**Fig. 5:**
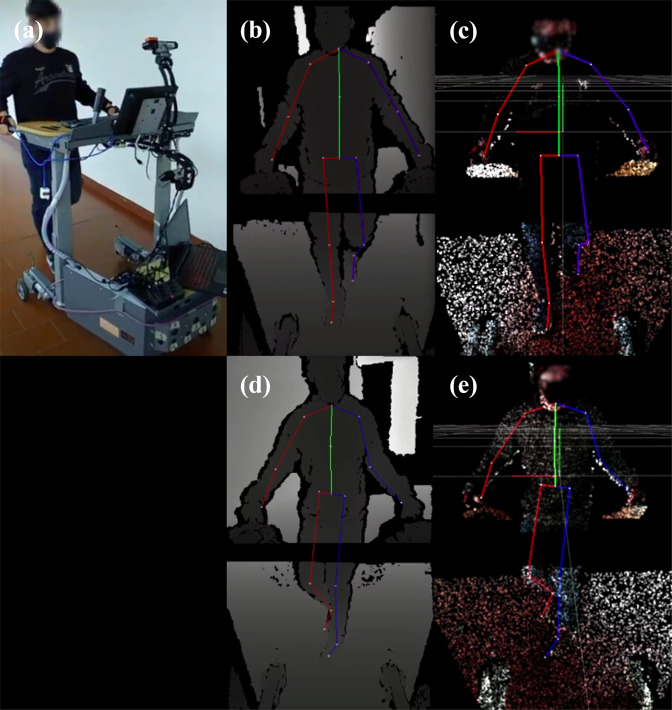
Samples of processed data from a forward scenario (upper) and cornering scenario (lower): (**a**) Outside view of the acquisition setup taken from a smartphone; (**b**) and (**d**) Depth camera frame overlaid with aligned 2D skeleton; and (**c**) and (**e**) 3D point cloud overlaid with aligned 3D skeleton. Data from the upper and lower cameras are related through the extrinsic transformation and displayed together.

Additionally, a visual inspection between the data from both cameras and the Xsens model was performed using the aligned skeleton data over a sequence of depth frames. This is illustrated in Fig. 5, which presents random samples of the dataset for one participant. Figure 5b illustrates the 2D projection of the skeleton coordinates overlaid with the depth frame, and Fig. 5c illustrates the same projection but in 3D representation (point cloud). Although the errors observed for the extrinsic calibration procedure, the aligned skeleton match the human joints of the depth frame. For instance, in Fig. 5b, it is possible to observe in the depth frame that the participant’s left foot is starting the swing phase of the gait cycle, which can also be verified with the segments of the aligned skeleton. Figure 5d,e illustrate the same participant performing a left turn, being the former a 2D projection and the latter a 3D one. Once more, the aligned skeleton matches the human body depicted in the depth frames. Nevertheless, we should point out that this projection is not without error, so it will be advantageous to use the normalised coordinates, depending on the application and if it is not strictly necessary to correlate the joint coordinates with the depth images.

## Data Availability

This database is accompanied by a folder with all the scripts used to process, handle, visualize, and evaluate the data described (available in *PhysioNet*^32^ and GitHub^34^). All scripts are based on the Python programming language and, thus, open source. The code contains a permissive MIT license for unrestricted usage. The dataset has also been used on a related publication, to develop and evaluate deep learning based algorithms for patient pose estimation using the robotic walker^35^. The authors hope it can further contribute to the development and evaluation of classic or data-driven vision-based pose estimation algorithms, applications in human detection, joint tracking, and movement forecasting, and gait/posture metrics analysis targeting solutions for motor rehabilitation.
